# Growth and fatty acid composition of pikeperch (*Sander lucioperca* L., 1758) larvae under altered feeding protocol including the copepod *Apocyclops panamensis* (Marsh, 1913)

**DOI:** 10.1038/s41598-023-46988-y

**Published:** 2023-11-10

**Authors:** Laura Ballesteros-Redondo, Harry W. Palm, Hanno Bährs, Tobias Rapp, Marcus Stueeken, Alexander Wacker, Adrian A. Bischoff

**Affiliations:** 1 Department of Aquaculture and Sea-Ranching, University of Rostock, Justus-Von-Liebig-Weg 2, 18059 Rostock, Germany; 2 Aquacopa GmbH, Hoher Damm 25, 17194 Jabel, Germany; 3 Department of Aquaculture, Mecklenburg-Vorpommern Research Centre for Agriculture and Fisheries, Institute of Fisheries, Malchower Chaussee 1, 17194 Hohen Wangelin, Germany; 4 Department of Animal Ecology, Zoological Institute and Museum, University of Greifswald, Loitzer Str. 26, 17489 Greifswald, Germany

**Keywords:** Fatty acids, Gas chromatography

## Abstract

Alternative live feeds for small and sensitive fish early life stages such as pikeperch (*Sander lucioperca* L., 1758) can improve the larval quantity, quality and performance in aquaculture. Therefore, this study evaluated the cyclopoid copepod *Apocyclops panamensis* (Marsh, 1913) as live feed for pikeperch larviculture from day 11 post hatch (dph) in two independent experiments. In both experiments, pikeperch larvae had the highest specific growth rate (SGR) when they fed on *Brachionus plicatilis* until dph 11 and *A. panamensis* until dph 16–18. SGR was related to a decrease in total fatty acids (FAs), saturated FAs and monounsaturated FAs in pikeperch larvae, indicating their use as energy for growth. Within the polyunsaturated FAs, docosahexaenoic acid (DHA) increased in larvae fed with *A. panamensis* and coincided with the highest SGR suggesting that DHA is accumulated in larvae as structural FA. Our study demonstrated a suitable pikeperch larval fatty acid composition for growth after feeding *A. panamensis* compared with *Artemia* sp. from dph 11 until dph 16 and previously fed with *B. plicatilis*. Moreover, it highlighted the importance of the dietary PUFAs in pikeperch rearing, specifically of linoleic acid (LA) from dph 4 until dph 11 and of DHA from dph 11 onwards.

## Introduction

The use of live feed for small and sensitive fish larvae has increased in aquaculture. Nevertheless, the sole use of *Artemia* spp. must be reconsidered since it does not fulfil the nutritional requirements of some fish species like pikeperch (*Sander lucioperca* (L., 1758))^1^. Rotifers such as *Brachionus plicatilis* (Mueller, 1786)^2^ and *B. calyciflorus* Pallas, 1766^3^ have been successfully introduced to pikeperch larviculture. *B. plicatilis* in combination with *Artemia* sp.^2^ or the exclusive diet with *B. plicatilis*^4, 5^ seemed to be adequate until day post hatch (dph) 10. However, the use should be limited to this period, avoiding negative effects on growth and intestinal development^5^. Fish larvae afford many physiological changes in the early life cycle stages and thus, a suitable feed must be supplied along all larval stages to fulfil their nutritional needs^6^. Beyond dph 11, rotifers are too small and limit fast growth therefore, *Artemia* sp. is still in use^2, 7^. However, *Artemia* lacks important nutrients for fish like docosahexaenoic acid (DHA)^8^. Enriched *Artemia* is limited since it depends on fish oils to rise their long chain polyunsaturated fatty acid (LC-PUFA) levels^9^ and even enriched, they seem to fulfil poorly the larval nutritional requirements^10^. El Kertaoui et al.^11^, Hamza et al.^12^, Hamza et al.^13^ and Lund et al.^14^ have shown that phospholipids and LC-PUFAs such as DHA are essential for pikeperch larvae at later larval stage (dph 17 to 34) since they may nutritionally program the fish for further development^11, 15^. Thus, there is still the need to find the optimal live feed for pikeperch larviculture after dph 10 beyond the application of rotifers.

Freshwater copepods are part of the natural diet of pikeperch larvae and thus, might fulfil the nutritional requirements. They have a higher nutritional value than rotifers and *Artemia* spp. due to their high natural amounts of PUFAs, free amino acids and antioxidant pigments^16^. For these reasons, the use of copepods in aquaculture has increased. Copepods are popular for ornamental fishes^17^ and have shown promising results for halibut larvae (*Hippoglossus hippoglossus*)^18^, winter flounder larvae (*Pseudopleuronectes americanus*)^19^, Atlantic cod (*Gadus morhua*)^20^, fat snook (*Centropomus parallelus*)^21^ and ballan wrasse (*Labrus bergylta*)^15^. Despite some copepods having high-PUFA content with low-PUFA diets^22, 23^ for some copepods, it is essential to provide high-PUFA diets since enrichment techniques are not appropriate^10^.

Ballesteros-Redondo et al.^24^ evaluated the potential of *Apocyclops panamensis* (Marsh, 1913) as live feed for larviculture. When *A. panamensis* was fed with *Isochrysis galbana* at 0.5–1 10^5^ cells mL^-1^ per day, copepod culture seemed to be adequate in terms of their fatty acid composition (1.8–2.6% of DHA and DHA/EPA ratio of 2.5–2.9) to rear fish larvae^25^. However, *A. panamensis* had no advantage for pikeperch larvae between dph 4 -10 in comparison to *B. plicatilis*^4^. Peterka et al.^24^ found nauplii of cyclopoid copepods in the stomach of pikeperch larvae, and El Kertaoui et al.^11^ reported a need of 3.5% of EPA + DHA for pikeperch larvae, which coincides with the fatty acid composition of *A. panamensis*^24^. The authors hypothesized that *A. panamensis* is an adequate live feed organism as a second live feed organism following the application of rotifers and improving the fatty acid composition. The present study evaluates the effect of *A. panamensis* on pikeperch larval growth and fatty acids composition between dph 11–18 after fed with *B. plicatilis* or *Artemia* sp. from dph 4 to dph 10 and compares it with the use of *Artemia* sp. between dph 11–16.

## Methods

### Live feed

Zooplankton as well as microalgae were obtained from Aquacopa GmbH, Jabel, Germany, and were cultivated at the facilities of the University of Rostock. According to Ferreira et al.^27^, *Brachionus plicatilis* (Müller 1786) was fed with *Nannochloropsis* sp. and, according to Ballesteros-Redondo et al.^24^, *Apocyclops panamensis* was fed with *Isochrysis galbana*. *Artemia* eggs (ArtemioPur, JBL GmbH & Co. KG, Germany) were hatched and a maximum of 24 h old Artemia nauplii was fed to the larvae. The density of each zooplankton culture was measured daily to harvest the amount needed to feed the pikeperch larvae (see experimental diets below). Besides that, three samples of *B. plicatilis* (67,500 individuals per sample) and *A. panamensis* (210,000 individuals per sample) were taken 24 h after the last supply of microalgae. Furthermore, three samples of recently hatched *Artemia* sp. were collected (40,000 individual per sample). To collect the individuals of each zooplankton organism, each culture was filtered through a 50 µm net, the organisms were collected with a minimum of water content in glass vials for subsequent lyophilisation. Afterward, samples were weighed and an amount between 1.3 and 1.9 mg dry weight (DW) of each sample was taken for fatty acid analyses. One mg DW for *B. plicatilis* corresponded to 1048 ± 186 individuals (ind.), for *Artemia* sp. 296 ± 36 ind. and for *A. panamensis* 4369 ± 533.

### Experimental set-up

Two independent experiments took place in October 2020 and March 2022.

**Figure 1:**
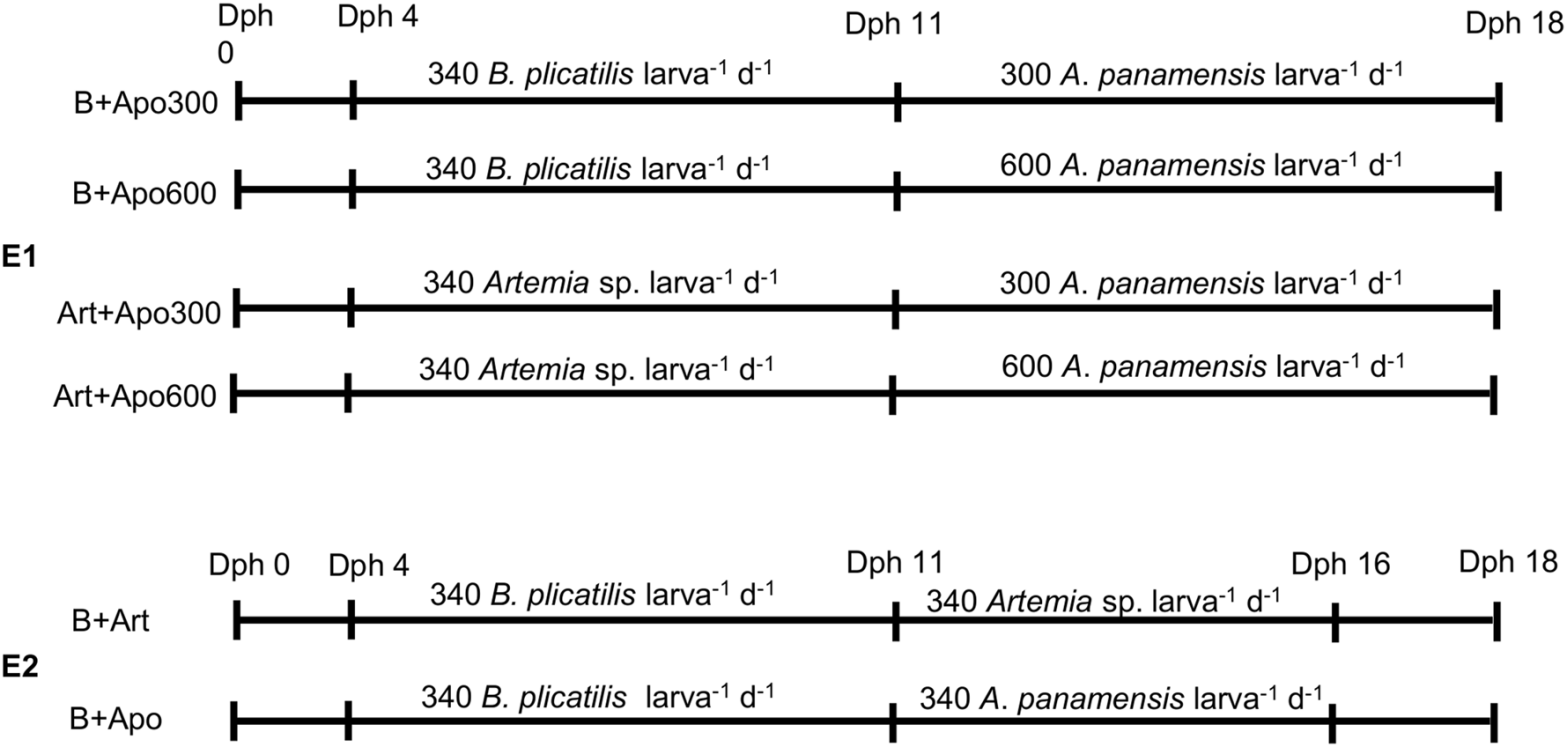
Feeding protocols applied in E1 (above) and E2 (below).

The first experiment (E1) was performed with fertilized pikeperch eggs from INAGRO, Belgium, transported cooled (< 10 °C) and brought to the experimental facilities of the University of Rostock, Germany. Upon arrival, the temperature was slowly raised and at a water temperature of 12 °C the eggs were transferred to an incubator. Within the next 48 h, the water temperature was continuously increased until 14 °C was reached. Three days after the transfer to the incubator, pikeperch larvae hatched. Larvae hatched within 24 h were stocked in 43 L tanks at 16 °C. Pikeperch larvae were maintained in a recirculating aquaculture system (RAS), including water treatment (mechanical and biological filtration as well as UV light treatment) under a light regime of 16L:8D, salinity of 0ppt, constant temperature and oxygen concentration. Two tanks with 43 L contained larvae at a density of 50 individuals per litre. While one tank was fed with *B. plicatilis* according to Ballesteros-Redondo et al.^4^, the other tank was fed with *Artemia* sp. (ArtemioPur, JBL GmbH & Co. KG, Germany). Both were fed from 4–10 dph three times per day (09:00, 12:00 and 15:00). Sixteen floating sub-units of 1 L were operated in parallel in two further 43 L tanks in the same recirculation system, arranged in four groups (each 4 replicates). On dph 10 after the last feeding time, larvae were stocked into the sub-units. Eight sub-units contained larvae fed previously with *B. plicatilis* and the other 8 sub-units larvae fed previously with *Artemia* sp. Four sub-units each were stocked with 25 larvae L^-1^, which were fed with 600 *A. panamensis* ind. larva^-1^ day^-1^, and the other sub-units with 50 larvae L^-1^ and fed with 300 ind. larva^-1^ day^-1^ from dph 11 until dph 18 (Fig. 1).

For the second experiment (E2) pikeperch larvae were obtained directly from the Pikeperch facility of the Mecklenburg-Vorpommern Research Centre for Agriculture and Fisheries in Hohen Wangelin (Mecklenburg-Western Pomerania, Germany). Larvae were transported at 15ºC to the experimental facilities of the University of Rostock at an age of 0dph and stocked into the experimental tanks at 16ºC. Pikeperch larvae were maintained in the same RAS as during the first experiment. Larvae were stocked at a density of 50 larvae L^-1^ and fed 340 *B. plicatilis* larva^-1^ day^-1^ from dph 4 until dph 10. After last feeding at dph10, again 50 larvae L^-1^ were stocked in 6 floating sub-units. As mortality increased during the first experiment from dph 16, in the second experiment the larvae were fed from dph 11 until dph 16 in two groups, one with *A. panamensis* and the other with *Artemia* sp. at 340 ind. larva^-1^ day^-1^ (Fig. 1). The period from dph 0–10 was analysed in E1 to monitor the effect of the first feeding period on the second period (dph 11–18) and in E2 to have the reference to compare with E1.

### Data collection and analyses

The physicochemical water parameters temperature, oxygen, and pH were monitored daily. Water samples were taken daily for subsequent analyses of dissolved nitrogen compounds ammonium (NH_4_^+^), nitrite (NO_2_^−^), nitrate (NO_3_^−^), orthophosphate (PO_4_^3−^) using an auto-analyser (Gallery Automated Photometric Analyzer Thermo Fisher Scientific, Waltham, MA, USA). In E1, larvae were reared at a temperature of 16.4 ± 0.4 °C, oxygen saturation of 103.8 ± 1.2% and pH = 8.7 ± 0.1, ammonium 0.58 ± 0.02 mg L^-1^, nitrite 0.07 ± 0.02 mg L^-1^, nitrate 11.7 ± 1.5 mg L^-1^, and phosphate 0.47 ± 0.29 mg L^-1^. In E2, larvae were reared at a temperature of 16.5 ± 0.5 °C, oxygen saturation of 105.7 ± 1.3% and pH = 8.3 ± 0.3, ammonium 0.39 ± 0.07 mg L^-1^, nitrite 0.35 ± 0.38 mg L^-1^, nitrate 20.9 ± 1.4 mg L^-1^, and phosphate 2.70 ± 0.48 mg L^-1^.

In addition, we performed daily siphoning of the bottom and removal of the dirt, dust and lipid layer at the water surface. Survival rate (E1 N = 4 and E2 N = 3) was calculated from dph 11, when the feeding with *A. panamensis* started, by counting recorded dead larvae from siphoning every day as follows:

1 $$Survival\left( \% \right) = \left[ {\left( {N_{i} - T_{D} } \right)100} \right]/N_{i}$$

where N_i_ is the initial number of larvae and T_D_ the total number of dead larvae found, cumulated over the experimental days.

All methods were carried out in accordance with German guidelines and regulations.

The experiments with pikeperch larvae were conducted within the German Animal Welfare Act guidelines and were approved by the authorities, in our case the Mecklenburg-Western Pomerania State Office for Agriculture, Food Safety and Fisheries, based in Rostock. The authors complied with the ARRIVE guidelines. According to the animal experiment permit issued (permission number 7221.3–1.1–051/19), in E1, 30 larvae were taken at random at dph 0 and dph 11 in each treatment. At dph 18, 7 larvae from each replicate were taken. In E2, 30 larvae were taken at random at dph 0, 4 and 11. At dph 16, 7 larvae from each replicate were taken. In both experiments, larvae were first cooled down until 12 °C, and subsequently until 0 °C to anesthetize them. Pictures for measurements were taken and larvae were killed by cutting the spinal cord and frozen for fatty acid analyses.

The total body length as well as yolk sac and oil droplet sizes were measured in both experiments under a stereo light microscope (SZX10 Olympus, Hamburg, Germany) connected to a UC30 digital camera (Olympus, Hamburg, Germany) and the software package cellSens Dimension 1.6 (Olympus Soft Imaging Solutions, Hamburg, Germany). Yolk sac volume and oil droplet volume were calculated according to Bischoff et al. (2018). Finally, we calculated the specific growth rate (SGR) [% d^-1^] (E1 N = 4, E2 N = 3) as follows assuming linear growth^4, 28^:

2 $$SGR = \left\{ {\left[ {\left( {L_{t} - L_{o} } \right)/L_{o} } \right]/t} \right\}100$$

where L_t_ and Lo represent the average length of the larvae at time t and time t = 0.

Fatty acid analyses of zooplankton as well as pikeperch larvae were performed at Greifswald University, in the Laboratory of Animal Ecology. The freeze-dried samples were transferred to extraction tubes, and dichloromethane:methanol (2:1, v:v) and nonadecanoic acid methyl ester as an internal standard was added to the samples. After ultrasonic treatment for > 5 s samples were kept under a nitrogen atmosphere at -25 °C until further analysis, which was done according to Wacker et al.^29^. Fatty acids were transesterified into fatty acid methyl esters (FAMEs) with methanolic HCl (Sigma-Aldrich Chemie, Taufkirchen, Germany)^30^^31^ and FAMEs were analysed by gas chromatography (6890N, Agilent Technologies, Böblingen, Germany) with helium as carrier gas^32^. For verification, mass spectra were recorded using a gas chromatograph-mass spectrometer (Pegasus 4D GC-TOFMS, LECO Instruments, Mönchengladbach, Germany).

Statistical analyses were performed by using the software IBM SPSS Statistics, Version 27. Normal distribution was tested using the Shapiro–Wilk Test. To analyse differences between means, Analysis of Variance (one-way ANOVA) or t-Test for independent samples was applied when normality was proven. Without normality, the Kruskal–Wallis or Mann–Whitney U test was applied. All significance levels α were set to 0.05. Data was reported as mean ± s.d.

## Results

### Live feed fatty acid composition

*B. plicatilis* had a total fatty acid (FA) content of 56.94 µg mg^-1^ DW consisting of 29.25% saturated fatty acids (SFAs), 48.50% monounsaturated fatty acids (MUFAs) and 22.23% polyunsaturated fatty acids (PUFAs). The SFA palmitic acid (16:0), the MUFA oleic acid (18:1) and the PUFA linoleic acid (LA, 18:2) were the most abundant single FA of each group (Table 1). *Artemia* sp. had the highest total FA concentration of 122.42 µg mg^-1^ DW, 18.00% SFAs, 37.4% MUFAs and 44.50% PUFAs. The SFA 16:0, the MUFA 18:1 and the PUFA linolenic acid (ALA, 18:3) were the most abundant single FA of each group (Table 1). *A. panamensis* had the lowest total FA concentration of 22.28 µg mg^-1^ DW, 40.00% SFAs, 8.90% MUFAs and 50.90% PUFAs. The SFA 16:0, the MUFA 18:1 and the PUFA docosahexaenoic acid (DHA, 22:6) were the most abundant single FA of each group (Table 1).

**Table 1:** Fatty acids contents of zooplankton in [µg mg^-1^ DW, mean ± s.d.

C:D	*B. plicatilis*		*Artemia* sp.		*A. panamensis*	
14:0	1.87^a^	± 0.10	0.32^b^	± 0.08	0.32^b^	± 0.17
15:0	0.12	± 0.04	0.01	± 0.03	0.01	± 0.01
16:0	10.49^a^	± 0.68	13.15^a^	± 1.46	5.63^b^	± 1.00
18:0	2.74^a^	± 0.36	7.29^b^	± 1.01	2.52^a^	± 0.21
20:0	0.06	± 0.10	0.07	± 0.06	0.04	± 0.07
22:0	1.39	± 0.24	1.39	± 0.64	0.45	± 0.20
16:1 sum	9.43	± 0.49	6.53	± 0.64	0.75	± 0.19
18:1 sum	16.50^a^	± 1.75	37.91^b^	± 3.18	1.21^c^	± 0.60
20:1 sum	1.64	± 0.20	1.32	± 0.63	0.01	± 0.02
22:1	0.05	± 0.09	0.00	± 0.00	0.01	± 0.02
18:2 n-6	6.43^a^	± 0.14	5.83^a^	± 0.72	0.66^b^	± 0.23
18:3 n-6	0.15	± 0.17	0.00	± 0.00	0.00	± 0.00
20:2 n-6	0.34	± 0.35	0.00	± 0.00	0.00	± 0.00
20:3 n-6	2.00	± 0.25	0.17	± 0.29	0.01	± 0.02
16:3 n-3	0.72	± 0.09	0.78	± 0.13	0.88	± 0.10
16:4 n-3	0.66^a^	± 0.16	0.96^b^	± 0.08	0.45^a^	± 0.07
18:3 n-3	0.90^a^	± 0.19	38.04^b^	± 4.04	0.91^a^	± 0.43
18:4 n-3	0.02^a^*	± 0.04	2.55^b^*	± 0.23	0.22^ab^*	± 0.10
20:3 n-3	0.00^a^*	± 0.00	0.40^b^*	± 0.32	0.00^a^*	± 0.00
20:4 n-3	0.02	± 0.04	0.23	± 0.22	0.01	± 0.02
20:5 n-3	1.28^a^	± 0.19	5.48^b^	± 0.76	1.61^a^	± 0.30
22:5 n-3	0.15	± 0.26	0.00	± 0.00	0.26	± 0.22
22:6 n-3	0.00^a^	± 0.00	0.00^a^	± 0.00	6.33^b^	± 0.77
SFA	16.66^a^	± 0.80	22.22^b^	± 2.21	8.96^c^	± 1.27
MUFA	27.62^a^	± 2.06	45.76^b^	± 3.05	1.98^c^	± 0.79
n-6	8.91^a^	± 0.81	6.00^b^	± 0.71	0.67^c^	± 0.25
n-3	3.75^a^	± 0.69	48.45^b^	± 5.24	10.66^a^	± 1.78
PUFA	12.66^a^	± 1.48	54.45^b^	± 5.94	11.34^a^	± 2.03
DHA/EPA	0.00^a^	± 0.00	0.00^a^	± 0.00	3.97^b^	± 0.29
Total FA	56.94^a^	± 1.43	122.42^b^	± 11.10	22.28^c^	± 4.00

“C” defines the number of carbon atoms and “D” the number of double bonds in the carbon chain.

The superscript a, b and c represent significant differences after ANOVA or Kruskal–Wallis test (*) (N = 3). Data reported as mean ± s.d.

### Pikeperch larvae survival

In E1, the survival rate until dph 16 was above 50% for all diets, for B + Apo300 72.0 ± 6.3%, B + Apo600 82.0 ± 7.7%, Art + Apo300 66.5 ± 6.8%, and Art + Apo600 59.0 ± 19.1%, and decreased until dph 18 to 7.5 ± 5.5% in B + Apo300, to 44.0 ± 3.3% in B + Apo600, to 32.0 ± 11.8% in Art + Apo300 and to 38.0 ± 10.1% in Art + Apo600. There were no significant differences in survival (Kruskal–Wallis *p* = 0.224, N = 4). In E2, the survival rate until dph 16 was 94 ± 2.8% for larvae fed with *Artemia* sp. and 87.9 ± 8.8% for B + Apo. No significant difference was found (t-Test *p* = 0.175, N = 3).

### Total body length and SGR

**Figure 2:**
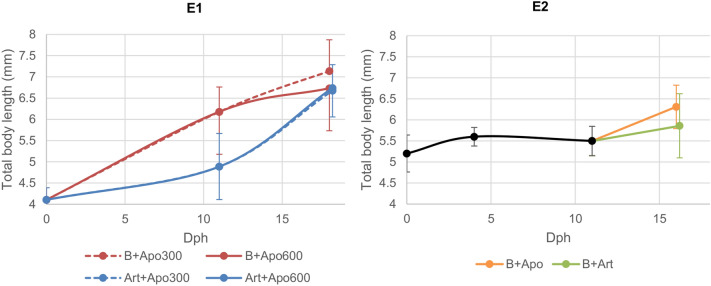
Total body length ± s.d. (mm) at the different days post hatch (dph) in experiment 1 (E1) and in experiment 2 (E2) with the different feeding protocols. Lines until dph 11 represent initial diet and different colour are different live feeds. Bifurcation at dph 11 shows change in diet. From dph 11, different colours are different live feeds and, in E1, continues or discontinuous lines are different quantities of feed.

In E1, larvae at dph 1 had a yolk sac volume of 2.05 ± 0.75 mm^3^ and a length of 4.11 ± 0.28 mm. After the feeding protocol with *B. plicatilis,* the larvae reached a length of 6.18 ± 0.58 mm at dph 11 while after being fed with *Artemia* sp. the larvae were 4.89 ± 0.78 mm long. The SGR in this period was 5.04 ± 1.42% d^-1^ for larvae fed *B. plicatilis* while the larvae fed with *Artemia* sp. reached 1.90 ± 1.89% d^-1^. At dph 18 larval total body length for B + Apo300 was 7.13 ± 0.71 mm, for B + Apo600 6.87 ± 0.16 mm. Total body length reached 6.67 ± 0.10 mm for Art + Apo300 and 6.74 ± 0.10 mm for Art + Apo600. There was no significant difference in length at the end of the experiment (ANOVA *p* = 0.092, N = 4) (Fig. 2). After the 7 days of exclusive feeding *A. panamensis*, SGR for B + Apo300 was 1.50 ± 0.20% d^-1^, for B + Apo600 1.12 ± 0.26% d^-1^, for Art + Apo300 3.64 ± 0.20% d^-1^ and for Art + Apo600 3.79 ± 0.19% d^-1^. Statistical differences were found between larvae previously fed with *B. plicatilis* and fed with *Artemia* sp. (ANOVA *p* < 0.001, N = 4). The SGR for the complete period (from 0 to 18 dph) was not significantly different among the groups B + Apo300 (4.33 ± 0.17% d^-1^), B + Apo600 (3.96 ± 0.23% d^-1^), Art + Apo300 (3.67 ± 0.14% d^-1^) and Art + Apo600 (3.77 ± 0.14% d^-1^) (ANOVA *p* = 0.092, N = 4).

In E2, larvae at dph 0 had a yolk sac volume of 0.84 ± 0.20 mm^3^ and a length of 5.17 ± 0.44 mm. After the feeding protocol with *B. plicatilis,* the larvae at dph 11 were 5.47 ± 0.35 mm and the SGR in this period was 0.52 ± 1.12% d^-1^. At dph 16 larval total body length for B + Apo was 6.31 ± 0.11 mm and significantly longer than 5.86 ± 0.24 mm for B + Art (Fig. 2, t-test *p* < 0.001, N = 3). After this 5-day feeding period with *A. panamensis* and *Artemia* sp., SGR for B + Apo was higher compared to the treatment B + Art (2.94 ± 0.4% d^-1^ and 1.32 ± 0.86% d^-1^, respectively, t-test *p* = 0.012, N = 3). This difference in the SGR was present for the complete period (from 0–16 dph), with 1.42 ± 0.14% d^-1^ for B + Apo and 0.85 ± 0.30% d^-1^ for B + Art (t-test *p* = 0.002, N = 3).

### Fatty acids composition

**Figure 3:**
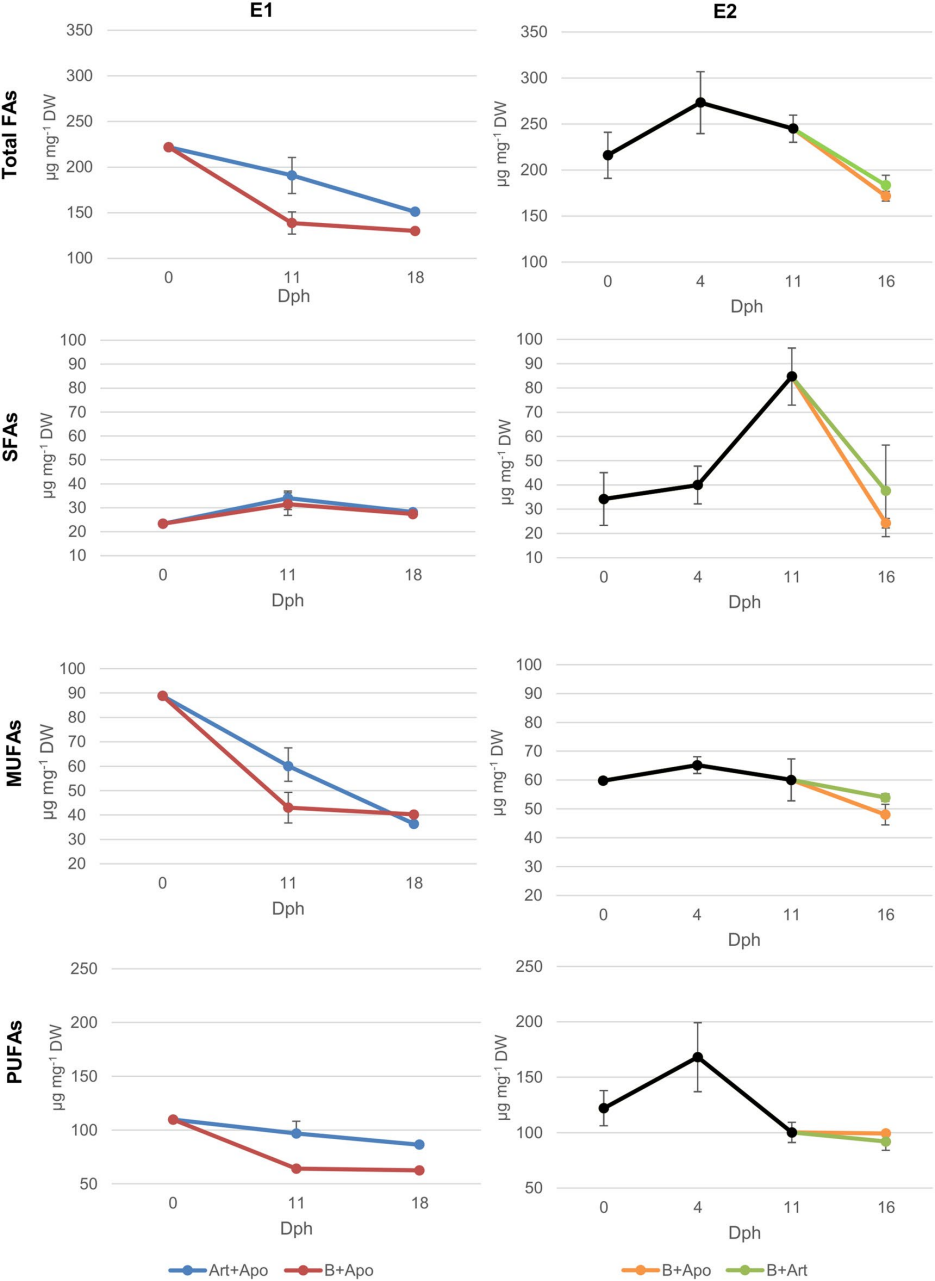
Fatty acids main group’s dynamics during both experiments (E1 and E2) under different feeding protocols (mean ± s.d.). For E2, black line until dph 11 is *B. plicatilis* diet. Bifurcation at dph 11 shows the change in diet.

In E1, larvae at dph 0 had a total FA content of 221.9 µg mg^-1^ DW, consisting of 10.54% SFAs, 40.02% MUFAs and 49.44% PUFAs. At dph 11, larvae fed with *B. plicatilis* had a total FA content of 138.6 µg mg^-1^ DW, consisting of 22.72% SFAs, 31.02% MUFAs and 46.25% PUFAs. Larvae fed with *Artemia* sp. (Art) had 190.9 µg mg^-1^ DW, with 17.86% SFAs, 31.48% MUFAs and 50.65% PUFAs. There was a significant difference in PUFAs in the larvae (t-Test *p* = 0.017) (Fig. 3). Both diets resulted in a similar composition of larval SFAs. Regarding MUFAS, larvae showed no significant difference in the content of palmitoleic acid (16:1). However, there was a significant difference in oleic acid content being lower in larvae fed *B. plicatilis* (t-Test *p* = 0.016) (SI- Table 1).

**Figure 4:**
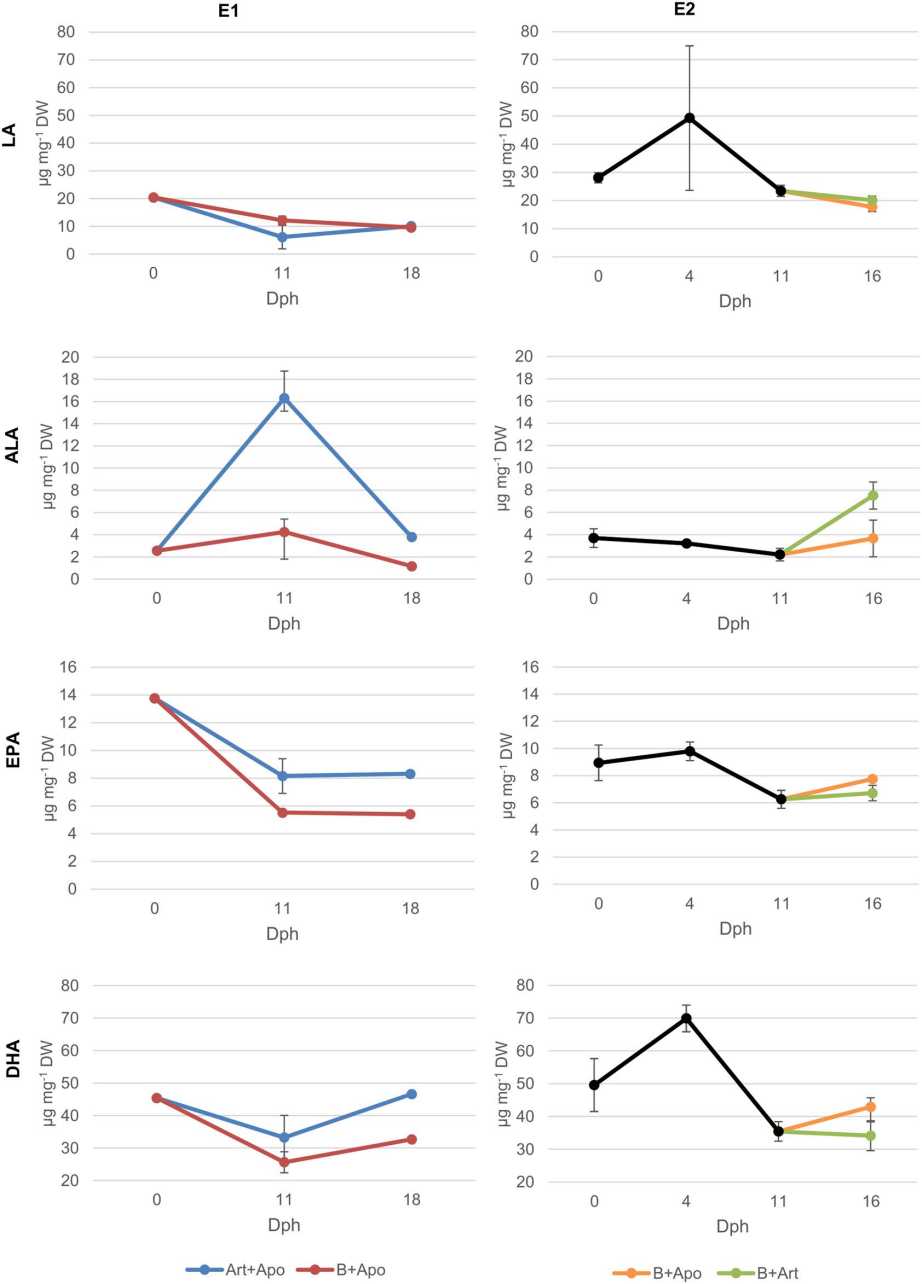
Important PUFAs dynamics during both experiments (E1 and E2) under different feeding protocols (mean ± s.d.). For E2, black line until dph 11 is B*. plicatilis* diet. Bifurcation at dph 11 shows the change in diet.

Larvae fed with *B. plicatilis* had also lower concentration of PUFAs (Fig. 3) (t-Test *p* = 0.016). In particular omega-3 (n-3) were lower (t-Test *p* = 0.007), for example ALA (t-Test *p* < 0.001), stearidonic acid (SDA, 18:4n-3) (t-Test *p* = 0.005), eicosatrienoic acid (ETE, 20:3 n-3) (t-Test *p* = 0.036), eicosatetraenoic acid (ETA, 20:4 n-3) (t-Test *p* = 0.011) and eicosapentaenoic acid (EPA, 20:5 n-3) (t-Test *p* = 0.041). Moreover, larvae showed no significant difference in DHA and in LA (Fig. 4), eicosadienoic acid (20:2 n-6), dihomo-gamma-linolenic acid (DGLA, 20:3 n-6) and DHA/EPA ratio. At dph 18, after the diet with *A. panamensis*, the feeding protocol B + Apo led to a total larval FA of 130.0 µg mg^-1^ DW, 21.07% SFAs, 30.92% MUFAs and 48.0% PUFAs. After feeding with Art + Apo, the total FA was 151.0 µg mg^-1^ DW with 18.67% SFAs, 24.11% MUFAs and 57.22% PUFAs (Fig. 3).

In E2, larvae at dph 0 had a total FA of 216.0 µg mg^-1^ DW, consisting of 15.83% SFAs, 27.69% MUFAs and 56.48% PUFAs. At dph 4 before feeding, larvae had a total FA of 273.2 µg mg^-1^ DW, 14.65% SFAs, 23.83% MUFAs and 61.53% PUFAs (Fig. 3). At dph 11, after feeding *B. plicatilis*, larvae had 244.9 µg mg^-1^ DW with 34.58% SFAs, 24.50% MUFAs and 40.90% PUFAs. At dph 16, after feeding protocol B + Art larvae showed a total FA of 183.4 µg mg^-1^ DW with 20.45% SFAs, 29.39% MUFAs and 50.10% PUFAs (Fig. 3). After B + Apo, the total FA was 171.5 µg mg^-1^ DW with 14.11% SFAs, 27.99% MUFAs and 57.90% PUFAs. There were no significant differences in SFAs and in particular C16:0 and stearic acid (18:0). Within the MUFAS, larvae fed B + Art diet showed significantly higher content of 18:1 (Mann–Whitney *p* = 0.05).

Larvae fed with B + Art had a lower concentration of PUFAs particularly in DGLA (Mann–Whitney *p* = 0.05), arachidonic acid (ARA, 20:4 n-6) (Mann–Whitney *p* = 0.05), docosapentaenoic acid (DPA, 22:5n-6) (t-Test *p* = 0.007) and EPA (Mann–Whitney *p* = 0.05) but a higher content of ALA (t-Test *p* = 0.034) (Fig. 4) and SDA (t-Test *p* = 0.038). Moreover, larvae showed no significant difference in DHA content and in DHA/EPA ratio (SI- Table 2).

## Discussion

Our study showed that *A. panamensis* is suitable as live feed for the rearing pikeperch larvae from dph 11 until dph 16. According to Ballesteros-Redondo et al.^4^, *B. plicatilis* was a suitable live feed for pikeperch larvae from dph 4 until dph 10. In the present study (E1), pikeperch larvae fed initially with *B. plicatilis* and followed by *A. panamensis* (B + Apo300 and B + Apo600) grew more (length of 6.18 mm on dph 11) than larvae fed initially with *Artemia* sp. (Art + Apo300 and Art + Apo600; length of 4.89 mm). Yanes-Roca et al.^2^ reported the same result on dph 11. Moreover, larvae fed first with *B. plicatilis* reached a higher SGR (5.04% d^-1^) from dph 0 to dph 11 compared with *Artemia* sp. (1.9% d^-1^). These SGRs of both treatments were higher than in Imentai et al.^33^ (1.37% d^-1^) and in experiment 1 of Ballesteros-Redondo et al.^4^. Our SGR of the larvae first fed with *B. plicatilis* and subsequently with *A. panamensis* exceeded the so far highest SGR of 3.0% d^-1^ when feeding solely 340 *B. plicatilis* per larva per day^4^. Consequently, the pikeperch larvae in the present study performed best in comparison to earlier studies and life feed combinations during this early life cycle stage.

With the increasing growth, energy and nutrient requirements of the pikeperch larvae from dph 11 onwards, larvae fed with the copepod *A. panamensis* had the highest survival rates of 72% and 82% until dph 16 (E1) for protocols B + Apo300 and B + Apo600, respectively. However, the mortality drastically increased from dph 16. This might indicate that *A. panamensis* should be best used until dph 16 (for a five-days feeding period). However, the mortality increase might be caused by the start of cannibalism^34^. On dph 18, after feeding *A. panamensis*, the longest size was achieved in protocol B + Apo300 (7.13 mm). Nevertheless, larval length is higher in other studies^2, 33^. Difference in breeders, genetics or initial larval quality makes the larval length on dph 18 difficult to compare. Thus, future studies should include more parameters such as initial fatty acid composition and larval weight^28^. Despite this, the SGR from dph 11 until dph 18 was lower for B + Apo300 and B + Apo600 compared with Art + Apo300 and Art + Apo600, which suggests a better larval development despite the initial supply with *Artemia* sp. This result indicated that larvae previously fed with *Artemia* sp. during the first 10 days were able to compensate a slower growth from dph 0 to dph 11 by feeding with *A. panamensis* afterwards. Nevertheless, B + Apo300 had a high SGR of 4.33% d^-1^ (dph 11–18), even higher than SGR data by Ballesteros-Redondo et al.^4^ and Imentai et al.^35^. Therefore, *A. panamensis* supplied an adequate level of energy and nutrient supply for the pikeperch larvae and was consequently well suitable to obtain an adequate larval development, independent of the feeding protocol B + Apo300, B + Apo600, Art + Apo300, and Art + Apo600. Nevertheless, our data showed the importance of including the growth data for the different live feeds since similar results with different initial live feeds might make the growth process of the larvae up.

Despite a suitable *B. plicatilis* supply and adequate water quality in E2, the larval growth was low during the first few days. On dph 11, the total body length (5.47 mm) was still similar to the initial length (5.17 mm). The total FA contents until dph 11 almost did not decrease as in normally developing larvae or in starving larvae^1^. The larvae did not consume their FA reserves (fed on *Brachionus*) while the growth and survival rates were still low. However, from dph 11 onwards, the use of *A. panamensis* significantly improved larval performance compared to the use of *Artemia* sp. On dph 16, the survival rate for B + Art was slightly higher (94.0%) than in B + Apo (87.9%), the latter similar to B + Apo600 in E1 (82%). These survival rates were higher than in Imentai et al.^5^, who reported survival rates of 35 -68% on dph 16, and Yanes-Roca et al.^2^, who reported survival rates of 35–75% on dph 21. However, our calculated survival rates only considered dph 11 onwards to study the effect of *A. panamensis* as a live feed. Therefore, larval survival and growth rates should be reported at the change of live feed organism. On dph 16, the larvae fed with B + Art were smaller compared with B + Apo and thus, the SGR was significantly higher in B + Apo (2.95% d^-1^) compared with B + Art (1.32% d^-1^). The SGR for B + Apo was also higher compared with Imentai et al.^5^, who fed pikeperch larvae with different combinations of *B. plicatilis* and/or *Artemia* sp. They reported the maximum SGR of 2.41% d^-1^ between dph 11–17 (according to our own calculations). Consequently, our larval growth data demonstrate that *A. panamensis* had a distinctly positive effect on the growth of pikeperch larvae between dph 11 and dph 16 in comparison to *Artemia* sp. as live feed.

The applied *B. plicatilis* had lower total FAs and PUFAs contents than *Artemia* sp. per dry weight (Table 1). Consequently, the pikeperch larvae fed with *B. plicatilis* showed a lower amount in total FAs and PUFA contents than the larvae fed with *Artemia* sp. However, the highest SGR was found for the larvae fed with *B. plicatilis*, which especially contained a higher amount of LA than *Artemia* sp. (Table 1). LA seems to be a highly relevant FA in the diet for pikeperch, as suggested by Ballesteros-Redondo et al.^4^, Bischoff and Kubitz et al.^3^ and Yanes-Roca et al.^2^. Yanes-Roca et al.^36^ stated that pikeperch larvae might have the capacity to desaturate and elongate fatty acids with 18 carbons like LA to obtain DHA during the first 12 days of life. However, Reis et al.^37^ demonstrated that pikeperch larvae cannot biosynthesize DHA at dph 20. Recently, Perez and Reis et al.^38^ have shown that *B. plicatilis* esterifies C18 PUFAs into phospholipids. An increase in dietary polar lipids increased the growth rate of pikeperch and showed earlier digestive structure development^12^. Phospholipids in the diet might contribute to a better absorption and transport of long-chain fatty acids through enhanced lipoprotein synthesis^40^. This is supported by the fact that total FAs, MUFAs, PUFAs, EPA and DHA contents are lower in larvae fed with *B. plicatili*s which, at the same time, had the highest SGR, demonstrating that all these groups of FAs might have been used for growth and that the LA possibly as polar lipid plays a crucial role during these first days of larval development. Our results showed the importance of including the study of the lipids in form of neutral and polar lipids. Thereby growing larvae use up their larval storages from the yolk sac. With all their PUFA lipid storages, and by growing and increasing their body weight and by producing and accumulating non-lipid biomass, the relative content of PUFA decreases. In contrast, the slower-growing larvae (after feeding *Artemia* sp.) might just use up energy (carbohydrates and SFA) without growing due to a less appropriate diet thus, increasing their proportion of MUFAs and PUFAs. This suggests that *B. plicatilis* might have a suitable fatty acid composition in the adequate form of polar lipids for pikeperch larvae.

From dph 11 to dph 18 (E1), there was a higher decrease in PUFAs in larvae fed with more PUFAs Art + Apo (10.7%) than fed with B + Apo (2.7%). The use of *B. plicatilis* might have improved the absorption of the LC-PUFAs by the larvae. We therefore suggest that the first live feed might affect the future success of the larvae development although this effect was not shown by an improved SGR based on the larval length. This result highlighted the importance of measuring the survival and larval growth when changing live feed organisms. Moreover, including larval weight in future studies might allow us to detect the effect of the first live feed on the larval growth. There was an increase in DHA for all treatments in E1 after feeding *A. panamensis*. *A. panamensis* is characterised by its higher content of DHA in comparison with *B. plicatilis* and *Artemia* sp. (Table 1). This higher DHA content has already been reported in copepods^41^ and in particular for *Apocyclops* species (for *A. royi*^23, 42, 43^ and for *A. panamensis*^24^). Therefore, our data demonstrate that pikeperch larvae could ingest and digest *A. panamensis*, and consequently could utilize the supplied nutrients. This underlined the possibility of rearing pikeperch larvae from dph 11 until dph 18 with this marine copepod species. However, since saltwater copepods do not survive long in freshwater, freshwater copepod species should be studied since they survive longer and might supply suitable nutrient composition to the freshwater fish species^3^.

*A. panamensis* also seemed to fulfill the nutritional requirements of the pikeperch larvae after first feeding on *B. plicatilis* better than feeding *Artemia* sp. In E2, the total FA concentrations, SFAs and MUFAs decreased more in B + Apo between dph 11 and dph 16, coinciding with a higher growth. When fish larvae grow, they require more energy. Both groups of fatty acids are used through the ß-oxidation to obtain adenosine triphosphate (ATP). This suggests that the pikeperch larvae used these groups of FAs as energy for growth. However, PUFAs decreased more in larvae fed with *Artemia* sp., which had a higher content of PUFAs than *A. panamensis* in our study (Table 1). This allows the conclusion that the PUFAs profile of *Artemia* sp. lacks important single fatty acids and that the FAs provided through *A. panamensis* were used. Although the total PUFAs decreased for both protocols, DHA only decreased in larvae fed with *Artemia* sp. as also shown by Yanes-Roca et al.^36^. The pikeperch larvae fed with *A. panamensis* instead increased their DHA content. While some FAs might have been used as energy for growth, in larvae fed with *A. panamensis* DHA accumulated, which is an important component used in fish retina^39^. Consequently, we demonstrate a better pikeperch larval fatty acid composition after feeding with *A. panamensis* compared with *Artemia* sp. Although *Artemia* sp. has more EPA, the larvae fed with B + Art increased their EPA content less than the larvae fed with B + Apo. This shows that the incorporation of these nutrients is more efficient when feeding *A. panamensis*. As mentioned before, phospholipids may enhance lipoprotein synthesis that improves absorption and transport of long-chain fatty acids^40^. Higher content of phospholipids in copepods compared to *Artemia* spp.^41^ might explain a better incorporation of EPA by the pikeperch larvae in our study. Consequently, our data demonstrate an improvement in pikeperch larviculture by the use of *A. panamensis* compared to *Artemia* sp.

Our pikeperch larvae kept during the experiments a minimum amount of 120 µg total FAs, 20 µg SFAs, 30 -40 µg MUFAs, 60 µg PUFAs, 4 µg EPA and 20 µg DHA per mg DW, and is definitively higher than those reported in starving larvae^1^. Furthermore, the content of PUFAs in our experiments was higher than those reported by Ballesteros-Redondo et al.^4^ and by Bischoff and Kubitz et al.^3^, which also reported lower SGR although the initial PUFAs contents were higher. Consequently, our study highlights the importance of the dietary PUFAs in pikeperch rearing, specifically of LA, from dph 4 until dph 11 and of DHA from dph 11.

It must be considered that the better performance of pikeperch larvae with *A. panamensis* occurred during the 5 consecutive days after an initial 10 days *B. plicatilis* feeding. This suggests an adequate timing and availability of both live feed organisms, making larviculture of pikeperch more complex. The high cost of copepod production is another constrain to be considered by the pikeperch hatcheries. Therefore, the economic viability and production efficiency of the combined *Brachionus* sp. and *A. panamensis* use must be further assessed.

Nevertheless, a more favourable dietary fatty acid composition will allow fish larvae to reach higher growth rates and thus allow the larvae to feed earlier and with less effort on bigger prey such as small fish. These other fish as prey items will then perfectly fit the nutritional requirements of the fish.

### Supplementary Information

Supplementary Tables.

## Data Availability

All data generated or analysed during this study are included in this published article (and its Supplementary Information files).
