# Supplementary material for: Growth and fatty acid composition of pikeperch (Sander lucioperca L., 1758) larvae under altered feeding protocol including the copepod Apocyclops panamensis (Marsh, 1913)
Source: Sci Rep. 2023 Nov 10;13:19574. doi: 10.1038/s41598-023-46988-y (PMC10638265; doi:10.1038/s41598-023-46988-y)
Supplement: Supplementary file 1 — Supplementary Tables. [file 41598_2023_46988_MOESM1_ESM.pdf]

GROWTH AND FATTY ACID COMPOSITION OF PIKEPERCH (*Sander lucioperca* L., 1758) LARVAE UNDER ALTERED FEEDING PROTOCOL INCLUDING THE COPEPOD *Apocyclops panamensis* (MARSH, 1913)

Laura Ballesteros-Redondo[1],*, Harry W. Palm[1], Hanno Bährs[2], Tobias Rapp[3], Marcus Stueeken[3], Alexander Wacker[4] and Adrian A. Bischoff[1]

[1]University of Rostock, Department of Aquaculture and Sea-Ranching, Justus-von-Liebig-Weg 2, 18059 Rostock, Germany laura.redondo@uni-rostock.de , harry.palm@uni-rostock.de , adrian.bischoff-lang@uni-rostock.de

[2]Aquacopa GmbH, Hoher Damm 25, 17194 Jabel, Germany baehrs@aquacopa.de

[3]Mecklenburg-Vorpommern Research Centre for Agriculture and Fisheries, Institute of Fisheries, Department of Aquaculture, Malchower Chaussee 1, 17194 Hohen Wangelin, Germany m.stueeken@lfa.mvnet.de , t.rapp@lfa.mvnet.de

[4]University of Greifswald, Department of Animal Ecology, Zoological Institute and Museum, Greifswald, Loitzer Str. 26, 17489 Greifswald, Germany alexander.wacker@uni-greifswald.de

*Correspondence: laura.redondo@uni-rostock.de

Supplementary information (SI)

SI-Table 1. Fatty acids contents in [μg mg^{-1} DW] of pikeperch larvae at dph 0, dph 11 and dph 18 in E1. A and b represent the significant differences only for samples at dph 11 (N=3 except for dph 18). Data was reported as mean ± s.d.

	DPH 0		DPH 11				DPH 18	
**C:D**			B		Art		B+Apo	Art+Apo
**14:0**	1.6	±0.6	0.7	±0.4	0.7	±0.1	0.1	0.2
**15:0**	0.2	±0.1	0.3	±0.1	0.2	±0.1	0.1	0.1
**16:0**	16.8	±3.7	20.6	±4.8	22.7	±2.8	17.5	17.4
**18:0**	4.3	±0.8	8.6	±0.8	9.6	±0.7	9.3	8.3
**20:0**	0.5	±0.0	0.5	±0.1	0.3	±0.0	0.3	0.3
**22:0**	0.0	±0.0	0.8	±0.4	0.5	±0.0	0.0	0.8
**24:0**	0.0	±0.0	0.2	±0.3	0.0	±0.0	0.0	1.0
**16:1 sum**	48.9	±15.3	15.3	±3.6	21.3	±6.8	15.8	14.6
**18:1 sum**	37.7	±8.6	25.6[a]	±4.3	37.4[b]	±2.7	23.1	21.4
**20:1 sum**	2.2	±0.7	1.7	±0.3	1.4	±0.2	1.3	0.4

**22:1**	0.0	±0.0	0.2[a]	±0.1	0.0[b]	±0.0	0.0	0.0
**24:1**	0.1	±0.1	0.2	±0.1	0.0	±0.1	0.0	0.0
**18:2 n-6**	20.4	±5.4	12.2	±1.8	6.1	±5.2	9.5	10.1
**18:3 n-6**	12.7	±3.2	4.4	±1.7	16.9	±10.9	4.3	4.8
**18:4 n-6**	0.5	±0.5	0.4	±0.2	0.2	±0.1	0.1	0.2
**20:2 n-6**	0.6	±0.1	1.0	±0.3	0.5	±0.2	0.4	0.5
**20:3 n-6**	1.8	±0.6	1.5	±0.1	1.0	±0.4	1.1	0.8
**20:4 n-6**	2.0	±0.3	2.0	±0.1	2.0	±0.2	2.2	2.5
**22:5 n-6**	0.8	±0.0	0.7	±0.0	0.8	±0.1	1.0	1.7
**16:3 n-3**	0.7	±0.2	1.6	±1.1	0.9	±0.1	0.7	1.3
**16:4 n-3**	0.3	±0.0	0.2	±0.3	0.6	±0.2	0.0	0.7
**18:3 n-3**	2.6	±0.5	4.2[a]	±1.4	16.3[b]	±2.9	1.2	3.8
**18:4 n-3**	1.4	±0.8	0.9[a]	±0.4	3.9[b]	±0.8	0.2	0.6
**20:3 n-3**	0.2	±0.1	0.6[a]	±0.1	1.0[b]	±0.1	0.1	0.4
**20:4 n-3**	1.3	±0.3	1.1[a]	±0.1	1.9[b]	±0.3	0.9	0.7
**20:5 n-3**	13.8	±2.0	5.5[a]	±0.1	8.2[b]	±1.5	5.4	8.3
**22:5 n-3**	5.2	±0.8	2.2	±0.3	3.3	±1.0	2.6	3.5
**22:6 n-3**	45.4	±4.3	25.6	±4.0	33.2	±8.4	32.7	46.6
**SFA**	23.4	±5.2	31.5	±5.8	34.1	±3.5	27.4	28.2
**MUFA**	88.8	±24.7	43.0	±7.7	60.1	±9.1	40.2	36.4
**n-6**	26.2	±7.1	18.1	±2.2	20.8	±4.5	14.8	15.9
**n-3**	83.4	±11.9	45.9[a]	±3.4	76.0[b]	±9.5	47.6	70.5
**PUFA**	109.7	±19.0	64.1[a]	±2.2	96.7$_b$	±14.0	62.4	86.4
**DHA/EPA**	3.3	±2.1	4.6	±0.7	4.0	±0.4	6.0	5.6
**TotalFA**	221.9	±48.9	138.6[a]	±12.2	190.9[b]	±19.7	130.0	151.0

“C” defines the number of carbon atoms and “D” the number of double bonds in the carbon chain.

SI-Table 2. Fatty acids contents in [µg mg^{-1} DW] of pikeperch larvae at dph 0, dph 4, dph 11, dph 16 and dph 18 in E2. A and b represent the significant differences and * show no normality (N=3). Data was reported as mean ± s.d.

	DPH 0		DPH 4		DPH 11		DPH 16				DPH 18	
**C:D**					B		B+Art		B+Apo		B+Apo	
**14:0**	1.7	±0.9	2.0	±0.7	3.9	±0.4	1.1	±1.4	0.4	±0.2	0.3	±0.3
**15:0**	0.6	±0.8	0.8	±0.7	2.7	±0.5	0.7	±1.2	0.1	±0.1	0.1	±0.2
**16:0**	21.6	±7.9	24.0	±4.7	45.8	±6.4	22.7	±12.3	15.6	±1.7	13.2	±3.2
**18:0**	9.2	±4.5	13.2	±3.5	31.9	±6.9	12.7	±7.7	8.2	±0.6	7.3	±1.7
**20:0**	0.3	±0.3	0.0	±0.0	0.3	±0.3	0.3	±0.5	0.0	±0.0	0.0	±0.0
**22:0**	0.8	±0.8	0.0	±0.0	0.0	±0.0	0.0	±0.0	0.0	±0.0	0.0	±0.0
**14:1**	0.1	±0.1	0.1	±0.1	0.5	±0.6	0.0	±0.0	0.0	±0.0	0.0	±0.0
**16:1 sum**	13.9	±0.5	13.9	±1.3	13.6	±2.9	11.1	±0.9	10.7	±1.5	11.9	±4.8
**18:1 sum**	45.0	±1.1	51.2	±3.4	45.9	±5.9	42.9[a*]	±1.0	37.2[b*]	±2.8	45.4	±16.5
**20:1 sum**	0.2	±0.4	0.0	±0.0	0.0	±0.0	0.0	±0.0	0.2	±0.3	0.0	±0.0
**22:1**	0.5	±0.4	0.0	±0.0	0.0	±0.0	0.0	±0.0	0.0	±0.0	0.0	±0.0
**18:2 n-6**	28.1	±2.2	49.3	±31.4	23.4	±2.4	20.1	±1.9	17.7	±2.0	23.8	±9.7
**18:3 n-6**	17.8	±0.9	21.5	±2.2	15.9	±3.5	11.9	±2.0	12.1	±0.3	14.8	±4.7
**18:4 n-6**	0.5	±0.6	0.1	±0.1	0.0	±0.0	0.0	±0.0	0.0	±0.1	0.0	±0.1
**20:2 n-6**	0.3	±0.5	0.0	±0.0	0.1	±0.3	0.5	±0.9	0.1	±0.0	0.1	±0.0
**20:3 n-6**	0.0	±0.0	0.0	±0.0	2.7	±2.3	1.5[a*]	±0.1	2.8[b*]	±0.8	3.1	±1.1
**20:4 n-6**	2.8	±0.4	2.9	±0.8	6.0	±3.9	2.8[a*]	±0.6	4.0[b*]	±0.2	4.0	±0.9
**22:5n-6**	0.9	±0.6	1.6	±0.7	1.1	±0.7	0.2[a]	±0.1	0.9[b]	±0.2	0.9	±0.2
**16:3 n-3**	1.5	±1.3	2.1	±0.8	2.3	±0.5	1.1	±0.5	1.7	±1.3	0.9	±0.1
**16:4 n-3**	1.2	±0.9	1.4	±0.2	0.8	±0.7	0.0	±0.0	0.4	±0.3	0.6	±0.1
**18:3 n-3**	3.7	±1.0	3.2	±0.2	2.2	±0.7	7.5[a]	±1.5	3.7[b]	±2.0	3.4	±0.5
**18:4 n-3**	1.1	±0.3	1.0	±0.3	0.8	±0.2	1.5[a]	±0.3	0.8[b]	±0.3	0.8	±0.1
**20:3 n-3**	0.2	±0.3	0.0	±0.0	0.0	±0.0	0.1	±0.1	0.2	±0.1	0.3	±0.1

**20:4 n-3**	1.7	±1.1	0.4	±0.4	0.0	±0.0	0.5	±0.4	0.4	±0.3	0.6	±0.5
**20:5 n-3**	8.9	±1.6	9.8	±0.8	6.3	±0.8	6.7[a*]	±0.7	7.7[b*]	±0.2	8.5	±2.4
**22:5n-3**	3.8	±0.7	5.0	±0.5	3.2	±0.4	3.4	±0.4	3.8	±0.3	4.7	±1.2
**22:6 n-3**	49.6	±9.9	69.9	±5.0	35.4	±3.6	34.1	±5.6	42.9	±3.4	53.5	±14.4
**SFA**	34.2	±13.3	40.0	±9.6	84.7	±14.4	37.5	±23.1	24.2	±2.4	20.9	±5.3
**MUFA**	59.8	±1.4	65.1	±3.5	60.0	±8.9	53.9	±1.6	48.0	±4.4	57.2	±21.3
**n-6**	50.4	±3.6	75.2	±32.9	49.2	±6.8	37.0	±5.1	37.7	±2.5	46.8	±16.3
**n-3**	71.7	±16.0	92.9	±5.3	51.0	±4.5	54.9	±4.7	61.5	±3.2	73.2	±18.8
**PUFA**	122.0	±19.5	168.1	±38.1	100.2	±11.2	91.9	±9.7	99.3	±1.3	120.0	±35.1
**DHA/EPA**	5.5	±0.2	7.2	±0.3	5.7	±0.2	5.1	±0.4	5.5	±0.4	6.3	±0.3
**TotalFA**	216.0	±30.7	273.2	±41.2	244.9	±18.0	183.4	±13.2	171.5	±6.4	198.1	±61.6

"C" defines the number of carbon atoms and "D" the number of double bonds in the carbon chain.
